# Prevalence of Diabetic Foot Ulcer and Associated Factors among Adult Diabetic Patients on Follow-Up Clinic at Jimma Medical Center, Southwest Ethiopia, 2019: An Institutional-Based Cross-Sectional Study

**DOI:** 10.1155/2020/4106383

**Published:** 2020-03-15

**Authors:** Daba Abdissa, Tesfaye Adugna, Urge Gerema, Diriba Dereje

**Affiliations:** ^1^Department of Biomedical Sciences (Clinical Anatomy), College of Medical, Sciences, Institute of Health Sciences, Jimma University, Ethiopia; ^2^Department of Biomedical Sciences (Medical Biochemistry), College of Medical, Sciences, Institute of Health Sciences, Jimma University, Ethiopia; ^3^Department of Biomedical Sciences (Medical Physiology), College of Medical, Sciences, Institute of Health Sciences, Jimma University, Ethiopia

## Abstract

**Background:**

Diabetic foot ulceration is a devastating complication of diabetes mellitus and is a major source of morbidity and mortality. So far, there are few published data on diabetic foot ulcers and its determinants among diabetic patients on follow-up at Jimma Medical Center. Hence, the aim of this study was to assess the prevalence of diabetic foot ulcer and its determinants among patients with diabetes mellitus at Jimma Medical Center.

**Methods:**

A hospital-based cross-sectional study was conducted from June 1 to August 30, 2019, and systematic random sampling technique was applied. The total number of study subjects who participated in the study was 277. Data were collected using an interview-administered structured questionnaire. Data were entered into EpiData version 3.1 and exported to SPSS version 20 software for analysis. Analysis was done using descriptive statistics and logistic regression. A variable having a *p* value of <0.25 in the bivariate model was subjected to multivariate analysis to avoid confounding the variable's effect. Adjusted odds ratios (AOR) were calculated at 95% confidence interval and considered significant with a *p* value of <0.25 in the bivariate model was subjected to multivariate analysis to avoid confounding the variable's effect. Adjusted odds ratios (AOR) were calculated at 95% confidence interval and considered significant with a

**Result:**

The mean of age of participants was 50.1 ± 14.19 years. More than three-fourths of participants (82.7%) were type 2 DM. The mean duration of diabetic patients was 6.00 ± 5.07 years. The prevalence of diabetic foot ulcer was 11.6% among study participants. According to multivariate logistic regression analysis, previous history of ulceration (AOR = 5.77; 95% CI: 2.37, 14.0) and peripheral neuropathy (AOR = 11.2; 95% CI: 2.8, 44.4) were independent predictors of diabetic foot ulcer.

**Conclusion:**

The prevalence of diabetic foot ulcer was 11.6%. Previous history of ulceration and peripheral neuropathy were associated with diabetic foot ulcer. The health care providers are recommended to thoroughly give emphasis during follow-up of patients who had previous history of ulceration and peripheral neuropathy in order to decrease the occurrence of diabetic foot ulcer.

## 1. Introduction

Diabetes has reached epidemic proportions worldwide. The International Diabetes Federation (IDF) estimates 425 million people living with DM worldwide in 2017, estimated to rise to 628 million by 2045. Sub-Saharan Africa is currently enduring the heaviest global burden of diabetes [1, 2].

Diabetic foot disease (DFD) is one of the diabetic complications associated with major morbidity, mortality, and reduced quality of life and is the most serious complication of diabetes mellitus [3, 4]. The incidence of DFD is still rising [5]. According to the international consensus on diabetic foot, a foot ulcer is defined as a full-thickness wound below the ankle in a diabetic patient, irrespective of duration [6].

The International Diabetes Federation estimates that at least one limb is lost due to DFU somewhere in the world every 30 seconds [7]. DFU is the most common cause of hospitalization in diabetic patients and also has significant socioeconomic impact [8, 9]. It is estimated that a person with diabetes has a 25% lifetime risk of developing DFU [10]. Patients with DFU have a greater than twofold increase in mortality compared with nonulcerated diabetic patients [11]. Five-year mortality rates after ulceration were around 40% [3]. Furthermore, the DFD and its long-term sequelae account for direct medical expenditures and lengthy periods of disability [12] .

According to a systematic review in 2017, the prevalence of foot ulcers among diabetic patients ranges from 3% to 13% globally [13]. In Africa, with constrained resources, the prevalence of DFU is higher. In sub-Saharan Africa, the burden of DFU is increasing due to late diagnosis, poor awareness among patients, and poor access to health care [13, 14].

DFU is preventable, and frequency of lower limb amputations can be lowered by 49-87% by preventing the development of DFU. Evidence in the literature suggests that the early detection and treatment of diabetic foot complications could reduce the prevalence of ulceration by 44% to 85% [15, 16]. Increased age, male gender, peripheral vascular disease, peripheral neuropathy, and renal disease were common risk factors for death after ulceration [3]. Patients at risk of developing DFU can easily be identified by clinical examination of the feet during follow-up [17]. Early screening of high-risk patients is important to prevent development of foot ulcers and its associated morbidity. To date, data regarding prevalence and factors related to foot ulcers among diabetic patients in Jimma are relatively few, and point prevalence varies in previous studies. So, the aim of this study is to solve this gap.

## 2. Methods

### 2.1. Study Area and Period

This study was conducted in Jimma Medical Center (JMC) which is located in Jimma town, Jimma zone, 355 km to the southwest of Addis Ababa, the capital city of Ethiopia. JMC is one of the largest hospitals in our country serving a very large catchment area in the Southwestern Oromia region. It gives different specialized clinical services including chronic follow-up for diabetes mellitus, hypertension, and other chronic illnesses. The study was conducted from June 1 to August 30, 2019.

### 2.2. Study Design

An institution-based cross-sectional study was conducted among adult diabetic patients on the follow-up clinic at Jimma Medical Center.

### 2.3. Population

The source population includes all adult diabetic patients on the follow-up clinic at JMC, while the study population was all adult diabetic patients who were under routine follow-up at the JMC during the study period.

### 2.4. Eligibility Criteria

Participants of age ≥ 18 years were included, and those who were seriously ill, gestational diabetic, diabetic patients who had traumatic ulcer, and clinically suspected of having Charcot foot were excluded.

### 2.5. Operational Definition

*Diabetic foot ulcer*: these are nontraumatic lesions of the skin on the foot distal to malleoli of a person who has diabetes mellitus.

*Clinically suspected patient with Charcot foot*: patients having DM for a long period of time and presented with a low level of sensation, swelling, and foot associated with midfoot collapse.

*Peripheral neuropathy*: this is defined as a patient with history version of MNSI questionnaire score ≥ 7, abnormal responses in the legs and/or if the lower extremity examination version of MNSI scores ≥2.5 in the legs [18].

*Foot deformities*: these are the presence of any of the following structural abnormalities in one or both feet: hammer toes, claw-toes, hallux valgus, prominent metatarsal heads, and amputations.

### 2.6. Sample Size Calculation and Sampling Procedure

The sample size was calculated using single population proportion formula by considering the prevalence of diabetic foot ulcer in Gondar, Ethiopia at 13.6% [19] at 95% confidence level and a 4% margin of error. It gives an initial sample size of 280. Since the source population of diabetic patients at the JMC clinic is less than 10,000, about 2500, by using the population correction formula for a finite population, the final sample size was calculated to be 251. By taking into consideration a 10% nonresponse rate, the final sample size was 277.

A systematic random sampling technique was employed to select study participants. The diabetes clinic runs twice weekly, and there were about 2500 diabetic patients on follow-up taken from the diabetes mellitus outpatient unit manager. These patients were our sampling frame, and the patients included in the sample were selected at every ninth interval. We got the interval by dividing the source population (2500) to the final sample size (277) and obtained nine. The first patient was selected randomly from the first ninth by a lottery method, and the next patient was interviewed and examined every ninth interval until the required sample was attained.

### 2.7. Data Collection Tool

Data were collected through a validated, pretested, and structured questionnaire which was developed after reviewing different literatures. The questionnaire contains sociodemographic factors, behavioral variables, clinical variables, and anthropometric measurements.

Clinical variables were taken from the patient record review, and anthropometric measurements were measured. Body weight was measured while wearing light clothes by an adjusted weight scale. Height was measured by meter, standing upright on a flat surface. Behavioral variables were assessed based on the WHO STEPwise approach for chronic disease risk factor surveillance [20]. BMI was calculated as kg/m^2^ to determine the nutritional status of the participant. Data collection was carried out by 2 BSC nurses and one medical intern with supervision of the principal investigator. After overnight fasting, blood samples were obtained for laboratory evaluation. The Michigan Neuropathy Screening Instrument was used to evaluate the presence of diabetic peripheral neuropathy (DPN) [21].

### 2.8. Data Analysis

The collected data were checked for completeness and coded. Then, the data were entered into EpiData version 3.1 and then exported to SPSS version 20.0 for analysis. Descriptive statistics such as frequencies, percentages, means, and standard deviations were computed as necessary. Bivariate and multivariate logistic regression models were used to determine the degree of association between the outcome and predictor variables. Variables having a *p* value of <0.25 in the bivariate model were subjected to multivariate analysis to avoid confounding the variables' effect. The goodness of fit of the multivariate model was checked with the Hosmer and Lemeshow test (*p* = 0.32). *p* value ≤ 0.05 was taken as statistically significant.

### 2.9. Data Quality Assurance

Data quality was ensured through standardized data collection materials, and questionnaires were thoroughly checked for completeness and consistency. To ensure the quality of data and cultural acceptance of the tool, pretests of data collection tools were carried out on 14 diabetes patients attending the Shenen Gibe hospital diabetic clinic prior to actual data collection. After analyzing pretest results, necessary modifications and corrections were made. Every day, the collected data was checked for completeness. Consequently, amendments and corrections were made.

### 2.10. Ethical Consideration

Ethical clearance was obtained from the Jimma University Institutional Review Board. A supportive formal letter was written to Jimma Medical Center. Data collection was done after permissions were obtained from hospital managers, and oral informed consent was obtained from the study participants to start data collection.

## 3. Result

### 3.1. Sociodemographic Characteristics of Participants

A total of two hundred and seventy-seven participants were involved in this study. More than half (165) of the respondents were males and the rest (112) were females. The mean age of the respondents was 50.1 ± 14.28 years. Regarding the marital status of the respondents, more than three-fourths (224, 80.9%) were married followed by singles (44, 15.9%) (Table 1).

### 3.2. Clinical and Behavioral Characteristics of Participants

Greater than three-fourths (82.7%) of the participants were type 2 DM. More than half (56.3%) of them were diagnosed with diabetes for less than 5 years, and almost one-third (31%) had no comorbid hypertension. A total of 189 (68.2%) of the study participants were in the normal category of BMI, whereas 44 (15.9%) of the participants were overweight. One hundred twenty-nine (46.6%) had diabetic peripheral neuropathy (Table 2).

### 3.3. Factors Independently Associated with Diabetic Foot Ulcer

Diabetic patients who had peripheral neuropathy were 11.2 times more likely to develop diabetic foot ulcer as compared with those who had no peripheral neuropathy (AOR = 11.2; 95% CI 2.8, 44.4; *p* = 0.001). Likewise, diabetic patients who had a history of ulceration were 5.77 times more likely to develop diabetic foot ulcer as compared with those who had no history of ulceration (*p* value = 0.00; AOR = 5.77; 95% CI 2.37, 14.0) provided other factors remain the same (Table 3).

## 4. Discussion

In the present study, the prevalence of diabetic foot ulcers among diabetic patients attending JMC was 11.6% (95% CI: 7.9, 15.5). This finding is in line with three independent studies done in Ethiopia, 13.6% in Gondar, 12% in Mekelle, and 14.8% in Arbaminch [19, 22, 23]. In addition, similar finding in North India (14.3%) and in Tanzania (15%) [24, 25]. However, this finding was lower than the study done in Addis Ababa, Ethiopia (31.1%) [26]; Telangana, India (16%) [27]; and Jordan (4.6%) [28]. The possible reason for such discrepancy might be due to difference in sample size used, study design, knowledge about foot self-care, health-seeking behavior, and health infrastructure of study participants.

In contrast, the finding of the current study is higher than a study conducted in Kenya which reported 4.6% [29]; Wollo, Ethiopia (4.4%) [30]; and Ghana which was 3.8% [31]. The possible difference might be due to difference in sample size, study design, and eligibility criteria.

The current finding demonstrated that participants who had peripheral neuropathy were 11.2 times more likely to develop diabetic foot ulcer than diabetic patients without peripheral neuropathy (AOR = 11.2; 95% CI: 2.8, 44.4). This result is consistent with prior studies [19, 27].This association is possibly because DPN promotes ulcer formation by causing loss of protective pain sensation, loss of pressure perception, and impairment of microcirculation [32, 33].

Furthermore, according to the current finding, participants who had a history of foot ulceration were 5.77 times more likely to develop diabetic foot ulcer than those without a previous history of foot ulceration (AOR = 5.77; 95% CI: 2.37, 14). The result is consistent with prior studies in Ghana and England [31, 34]. This association can be explained by biomechanical factors such as the degree of barefoot and in-shoe mechanical stress and the level of adherence to wearing prescribed footwear. In addition, it may be due to the fact that ulcer leads to microvascular dysfunction, macrovascular dysfunction, and peripheral nerve damage [35].

## 5. Conclusion

The prevalence of diabetic foot ulcer was 11.6% among study participants. Previous history of ulceration and peripheral neuropathy were independent predictors of diabetic foot ulcer. The health care providers are recommended to give emphasis during follow-up of patients who had a previous history of ulceration and manage the neuropathy thoroughly in order to decrease the occurrence of diabetic foot ulcer. In addition, future efforts should be directed toward educating both the healthcare professionals and patients about proper foot care.

### 5.1. Limitation of the Study

The duration of diabetes as measured in this study might not reflect the true duration of the disease, because the time since diagnosis and actual diabetes onset might precede diagnosis type 2 diabetes. Another limitation is the cross-sectional nature of the study which does not confirm the definitive cause and effect relation.

We did not asses the vascular status of our study population, so that we could not asses the prevalence of peripheral arterial disease.

## Figures and Tables

**Table 1:** Sociodemographic characteristics of patients with diabetes mellitus at JMC 2019, Jimma, Ethiopia.

Variables	Category	Number	Percentage
Sex	Male	165	59.6
	Female	112	40.4

Age (in years)	<30	32	11.6
	30 to 39	24	8.7
	40 to 49	72	26
	≥50	149	53.8

Marital status	Married	224	80.9
	Single	44	15.9
	Others^∗^	9	3.2

Education status	Illiterate	86	31
	Primary	119	43
	Secondary	34	12.3
	College and above	38	13.7

Occupational status	Housewife	84	30.3
	Farmer	87	31.4
	Employer	56	20.2
	Private worker	36	13
	Others^†^	14	5.1

Residence	Urban	86	31
	Rural	191	69

Income (Ethiopian birr)	<1000	83	30
	1000 to 1999	33	11.9
	2000 to 2999	64	23.1
	≥3000	97	35

^∗^Widowed and divorced; ^†^retired and unemployed.

**Table 2:** Clinical and behavioral characteristics of patients with diabetes mellitus at JMC 2019, Jimma, Ethiopia.

Variables	Category	Number	Percentage
Type of DM	1	48	17.3
	2	229	82.7

Duration of DM	<5 years	156	56.3
	5 to 10 years	73	26.4
	≥10 years	48	17.3

BMI	<18.5	29	10.5
	18.5 to 24.9	189	68.2
	25-29.9	44	15.9
	≥30	15	5.4

Alcohol intake	Current	33	11.9
	Former	25	89
	Never	219	79.1

Smoking	Current	14	5.1
	Former	41	14.8
	Never	222	80.1

Physical exercise	Active	115	41.5
	Inactive	162	58.5

Comorbid hypertension	Yes	86	31
	No	191	69

Fasting blood sugar	<200 mg/dl	204	26.4
	≥200 mg/dl	73	73.6

History of ulceration	Yes	90	32.5
	No	187	67.5

Peripheral neuropathy	Yes	129	46.6
	No	148	53.4

Foot deformity	Yes	97	35
	No	180	65

**Table 3:** Independent predictors of diabetic foot ulcer among diabetic patients at JMC 2019, Jimma, Ethiopia.

Variables	Category	Diabetic foot ulcer		Bivariate analysis		Multivariate analysis	
		Yes	No	*p* value	COR (95% CI)	*p* value	AOR (95% CI)
Age	<30	1	31	1	1	1	1
	30-39	2	22	0.409	2.8 (0.24, 33.04)	∗∗	
	40-49	9	63	0.167	4.4 (0.54, 36.5)	∗∗	
	≥50	29	129	0.133	4.8 (0.62, 37.2)		

Smoking	Current	8	23	0.023	2.91 (1.15, 7.33)	∗∗	
	Former	3	46	0.345	0.547 (0.156, 1.91)	∗∗	
	Never	21	176	1	1	1	1

Physical exercise	Active	10	105	1	1	1	1
	Inactive	22	140	0.21	1.65 (0.75, 3.63)	∗∗	

Peripheral neuropathy	Yes	27	95	0.00	8.53 (3.17, 22.9)	0.001^∗^	11.2 (2.8, 44.4)
	No	5	150	1	1	1	1

History of ulceration	Yes	23	67	0.00	6.78 (2.99, 15.41)	0.00^∗^	5.77 (2.37, 14.0)
	No	9	178	1	1	1	1

Alcohol intake	Current	1	32	0.136	0.21 (0.028, 1.62)	∗∗	
	Ex-drinker	3	22	0.911	0.93 (0.26, 3.31)		
	Never	28	191	1	1	1	1

Type of DM	T1DM	3	45	0.216	0.46 (0.13, 1.57)	∗∗	
	T2DM	29	200	1	1		

Foot deformity	No	14	166	1	1	1	1
	Yes	18	79	0.009	0.37 (0.175, 782)	∗∗	

^∗^Value statistically significant. AOR: adjusted odds ratio; COR: crude odds ratio; CI: confidence interval 1-reference. ^∗∗^Not statistically associated with diabetic foot ulcer.

## Data Availability

The original data of this study could be available for the third body only up on authors request.

## Abbreviations

AOR: Adjusted odds ratio
BMI: Body mass index
CI: Confidence interval
DM: Diabetes mellitus
DFD: Diabetic foot disease
DFU: Diabetic foot ulcer
COR: Crude odds ratio
JMC: Jimma Medical Center.
